# Benefits of Nutrients and Phytonutrients in Nuts and Legumes on Platelet Aggregation Through DNA Methylation

**DOI:** 10.1002/mnfr.70297

**Published:** 2025-10-27

**Authors:** Siwaphorn Chaimati, Jintana Sirivarasai, Nareerat Sutjarit

**Affiliations:** ^1^ Nutrition Unit Faculty of Medicine Ramathibodi Hospital Mahidol University Bangkok Thailand

**Keywords:** antiplatelet aggregation, DNA methylation, legumes, nuts, phytochemical, plant‐based diet

## Abstract

Platelets play a central role in hemostasis and thrombosis, but their hyperactivity is closely associated with an increased risk of cardiovascular diseases (CVDs). Appearing evidence emphasizes the importance of plant‐based diets, especially those rich in nuts and legumes, which are associated with modulating platelet function and reducing CVD risks. Nuts and legumes are abundant sources of nutrients, including mono/polyunsaturated fatty acids, fiber, vitamins, minerals, and bioactive compounds, such as polyphenols, flavonoids, and phenolic acids. These components, both nutrients and bioactive compounds, were reported to have antiplatelet aggregation properties. Furthermore, epigenetic mechanisms, particularly DNA methylation, regulate gene expression, including genes involved in platelet activation pathways, such as platelet endothelial aggregation receptor 1 (PEAR1), which are related to nutrients such as folate and methionine, contribute to one‐carbon metabolism, and bioactive compounds promote methylation patterns related to DNA methylation enzymes and reduced platelet hyperactivity. So, this review summarizes current findings and explores the potential association between nuts and legumes consumption and platelet aggregation (PA) through DNA methylation, emphasizing their role in cardiovascular health and the potential for targeted dietary interventions.

## Graphical abstract

Nuts and legumes contain both nutrients and bioactive compounds supporting cardiovascular health related to influencing DNA methylation. These foods are related to reducing oxidative stress, supplying methyl donors, and modulating genes involved in platelet function, such as PEAR1 and mitochondrial genes, in association with helping reduce inflammation and preventing blood clotting.

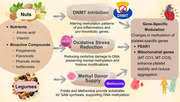

## 1 Introduction

Cardiovascular diseases (CVDs) remain the primary cause of death worldwide, approximately 17.9 million deaths annually, and 32% of global fatalities [1], with heart attacks and strokes accounting for 85% of all cases [2]. Atherosclerosis is a progressive inflammatory and metabolic condition that leads to plaque buildup, endothelial dysfunction, and thrombotic events [3]. Platelet aggregation (PA) plays an important role in thrombosis and vascular blockages by adhering to and aggregating at vascular injury sites [4, 5]. Platelets are rich in growth factors and cytokines that respond to signals to regulate biological processes such as inflammation, angiogenesis, and cell proliferation, contributing to various disease pathologies [6]. CVD prevention can be achieved by improving behavior and environmental risks, such as tobacco use, obesity, physical inactivity, excessive alcohol consumption, air pollution, and, especially, an unhealthy diet [1].

A plant‐based diet (PBD) is now widely recognized for reducing CVD risks. PBDs include variations like vegan, lactovegetarian, and ovovegetarian diets and can be categorized into healthy (e.g., whole fruits, vegetables, whole grains, legumes, and nuts) and unhealthy (e.g., fruit juices, refined grains, French fries, and chips) options [7]. PBD has been linked to reduced risks of obesity, metabolic syndrome, hypertension, and CVDs. A meta‐analysis found that PBDs were associated with lower blood pressure compared to animal‐based diets [8]. Similarly, a meta‐analysis of randomized controlled trials (RCT) revealed that, compared to an animal‐based diet, vegetarian diets significantly reduced systolic (−2.66 mmHg, *p* < 0.001) and diastolic blood pressure (−1.69 mmHg, *p* < 0.001) [9]. Remde et al. highlighted improved cardiometabolic outcomes and weight control due to lower trans and saturated fats and higher polyunsaturated fats in PBDs [10]. Kahleova et al. demonstrated that a 16‐week low‐fat vegan diet reduced fat mass and insulin resistance and increased insulin secretion [11]. Moreover, Tong et al. found that vegetarians and pescatarians had a 13% lower rate of ischemic heart disease but a 20% higher rate of strokes, particularly hemorrhagic strokes [12]. PBDs rich in nuts and legumes provide high fiber; vitamins (E and folate); minerals (potassium, calcium, magnesium); phytosterols; polyphenols; and unsaturated fatty acids [13]. Nuts are high in monounsaturated fatty acids (MUFAs) and polyunsaturated fatty acids (PUFAs), whereas legumes are low in fat; cholesterol‐free; and rich in fiber, protein, and essential nutrients (iron, copper, magnesium, manganese, zinc, and vitamin B) [14, 15]. Studies have highlighted their positive effects on metabolic diseases. Tharrey et al. found a lower cardiovascular mortality risk (HR: 0.60, *p* < 0.001) for those consuming nuts/seeds versus meat eaters (HR: 1.61, *p* < 0.001) [16]. Arabi et al.’s meta‐analysis revealed that walnut consumption (30–108 g/day) was associated with significantly lower triglyceride levels compared to control groups (isocaloric diets without walnuts or walnut‐fortified meals). The walnut group showed a mean reduction of −0.17 (95% CI: −0.32, −0.03) in triglyceride levels [17]. Furthermore, Ferreira et al. reported that high legume diets improved blood pressure, total cholesterol, LDL‐C, HDL‐C, and triglycerides due to their high MUFAs, PUFAs, and plant sterol content [18, 19].

Diets and epigenetics, which are connected to the mechanisms underlying several metabolic disorders, will be considered in future research investigations. Although PBD may impact global or gene‐specific DNA methylation by altering or supplying methyl donor chemicals or cofactors that control the metabolism of one‐carbon [20], no recent review articles have focused exclusively on nuts and legumes consumption, their effects on health, especially metabolic diseases, and their molecular mechanisms. This article summarizes the effects of nutrients and phytonutrients from nuts and legumes on PA through DNA methylation, one of the key mechanisms in the development of CVDs.

## 2 Molecular Mechanism in Platelet Aggregation and CVDs

### 2.1 Mechanism of Platelet Aggregation

**FIGURE 1:**
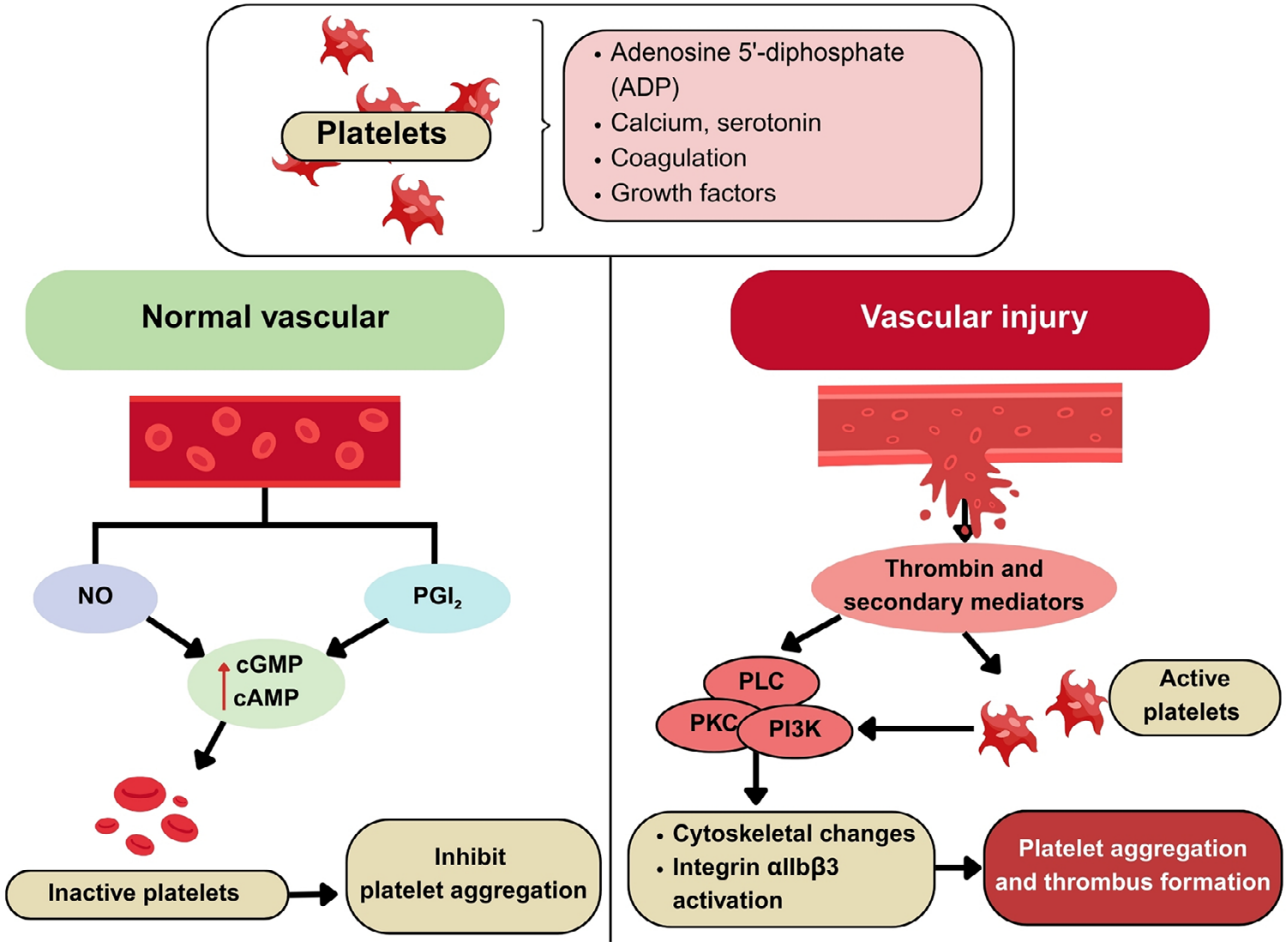
Platelet aggregation in normal and injured vascular (created with CAVA.com, by Siwaphorn Chaimati).

Platelets, which are small nucleus‐free blood cells, were initially identified as primary wound‐healing components through various activation mechanisms [21]. Platelets are produced in bone marrow by megakaryocytes. Their granules contain substances such as adenosine 5'‐diphosphate (ADP), calcium, serotonin, coagulation, and growth factors (Figure 1). Upon activation, platelets change shape, release granule contents, and cause aggregation and thrombus formation [22]. Platelets remain inactive in circulation due to nitric oxide (NO) and prostacyclin (PGI_2_) from the vascular endothelium, which increases cGMP and cAMP levels, activating protein kinases that inhibit PA. Upon vascular injury, exposure of the subendothelial matrix, mainly collagen, leads to platelet adhesion and activation. This process is amplified by thrombin and secondary mediators, engaging signaling pathways such as phospholipase C (PLC), protein kinase C (PKC), and phosphatidylinositide‐3‐kinase (PI3K). These pathways promote cytoskeletal changes, granule secretion, and integrin αIIbβ3 activation, which are essential for PA and thrombus formation (Figure 1). Negative feedback mechanisms, including immunoreceptor tyrosine‐based inhibition motif‐containing receptors, endothelial cell‐selective adhesion molecule, phosphatases, and receptor desensitization, regulate platelet activity to prevent excessive clotting. This balance ensures an effective response to vascular injury while minimizing the risk of occlusive thrombosis [23].

Platelets play multifaceted roles in disease pathophysiology, including (1) maintaining and regulating vascular tone by serotonin uptake during rest, serotonin, thromboxane, and prostaglandin release; (2) host defense (bacteria and virus phagocytosis/internalization, bacterial death, platelet microbicidal protein release, superoxide generation); and (3) inflammation (atherosclerosis, allergic asthma, kidney disease, plate‐let‐leukocyte interactions); and (4) hemostasis and thrombosis include adhesion, activation, spreading, secretion, aggregation, procoagulant activity, clot retraction, and tissue repair. They are crucial indicators of inflammation and are linked to CVDs and atherosclerosis, both of which are associated with type 2 diabetes. Platelets create thrombin, an agonist that plays a marked role in angiogenesis and inflammation [24].

### 2.2 PA Leads to CVDs

Platelets regulate inflammation, thrombosis, and hemostasis, all of which are crucial in vascular disorders. While essential for preventing bleeding, platelet overactivation contributes to diseases such as abdominal aortic aneurysm, myocardial infarction (MI), stroke, and atherosclerosis. By interacting with leukocytes and endothelial cells, platelets promote vascular inflammation and thrombosis, potentially leading to serious cardiovascular events [25]. By binding to oxidized low‐density lipoprotein, which activates platelets and accelerates the generation of foam cells, platelets may promote atherogenic vascular inflammatory processes. They can then attach to leukocytes and activate them. After promoting monocyte/macrophage infiltration and differentiation, chemokines are deposited on the inflammatory endothelium, which attracts leukocytes. These chemokines then attach to the endothelium, triggering an inflammatory response in endothelial cells. This process facilitates the recruitment of leukocytes to the endothelium through interactions with von Willebrand factor and the release of extracellular vesicles. In addition, by releasing inflammatory mediators that affect monocytes and neutrophils, directly promoting leukocyte recruitment and infiltration at the injury site, and inducing the release of neutrophil extracellular traps that can act as a substrate for both leukocyte recruitment and the coagulation reaction, platelets can support ischemic thromboinflammatory processes [26].

## 3 Nutrients and Phytochemicals in Nuts and Legumes

### 3.1 Nutrients in Nuts and Legumes

**FIGURE 2:**
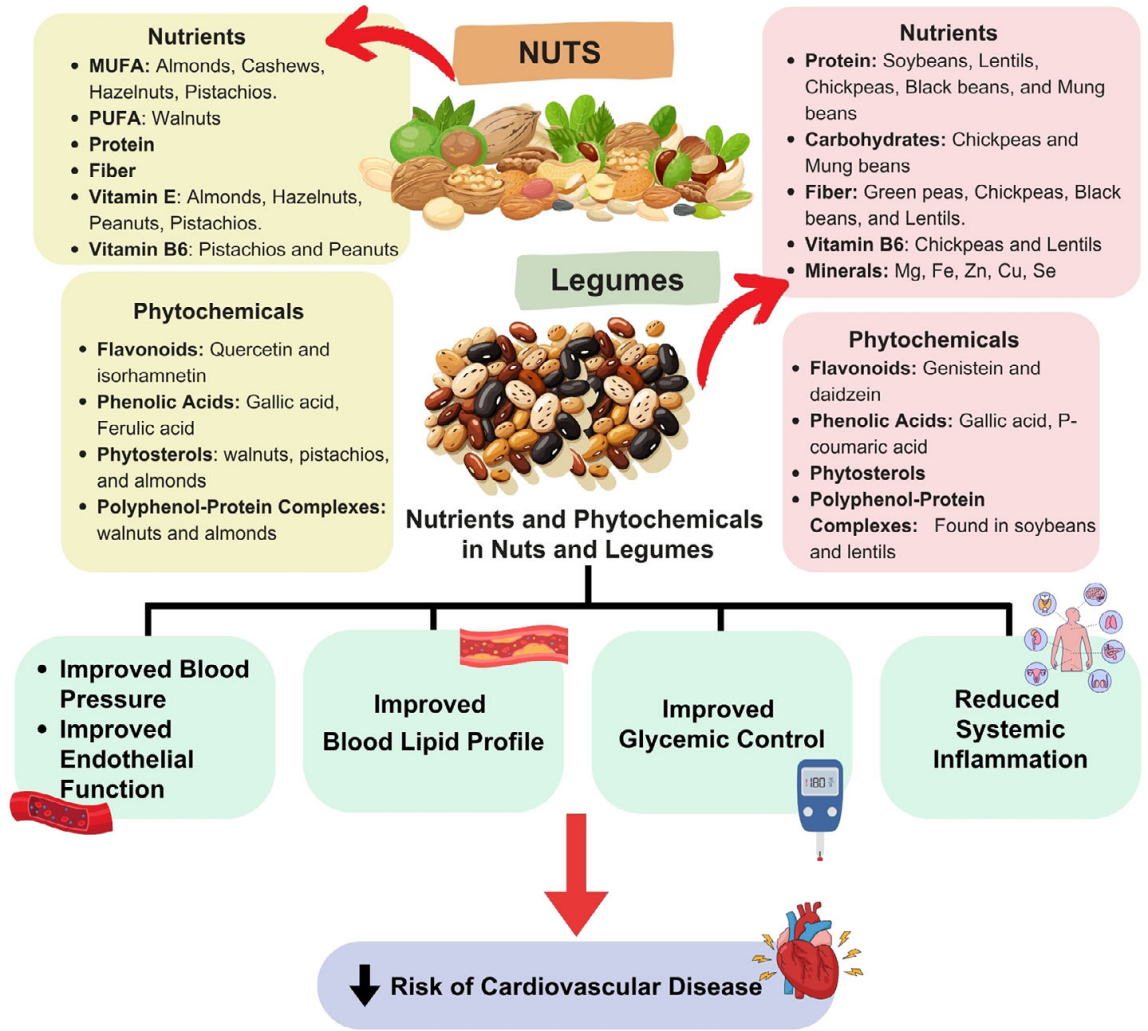
Nutrients and phytochemicals in nuts and legumes, and effects on cardiovascular health (created with CAVA.com, by Siwaphorn Chaimati).

Nuts include cashew nuts, hazelnuts, Brazil nuts, walnuts, almonds, pistachios, macadamias, and peanuts. These are considered nutritious foods in PBD due to their special nutritional value, and people worldwide are advised to consume them [27]. Nuts are eaten as snacks, sweets, or meals in Western and Asian nations. They can be eaten whole (roasted or fresh), in spreads (almond paste, peanut butter), as oils, or concealed in mixes, baked goods, ice creams, and commercial products [28]. Furthermore, nuts include soluble and insoluble fibers; protein; vitamin K; vitamin E; thiamine; folate; minerals (e.g., copper, magnesium, selenium, and potassium); antioxidants; phytosterol compounds; and xanthophyll carotenoids, as well as healthy fatty acid profiles that include MUFAs and PUFAs. These compounds can positively affect human health [27]. Legumes, or pulses, include various edible seeds such as kidney beans, black beans, chickpeas, lentils, and soybeans [15]. They are high in protein, fiber, and micronutrients, with low lipid content (except for soy) [29]. As a high‐protein, eco‐friendly food, legumes have been linked to a reduced risk of chronic diseases. Incorporated into dietary patterns such as the Mediterranean, DASH, low glycemic index, and high‐fiber diets, they offer notable health benefits. Legumes also feature prominently in national dietary guidelines (USDA, HHS, NHS) and cancer prevention recommendations (AICR, WCRFI) [14]. In Figure 2 illustrates the nutrients and phytochemicals found in nuts and legumes, and their effects on cardiovascular health. The effect of nuts and legumes consumption is related to reducing cardiovascular risk, including dyslipidemia, insulin resistance, hypertension, oxidative stress, and inflammation. Nuts are rich in healthy fats, MUFAs and PUFAs, while legumes have higher protein content. Both are also high in fiber and bioactive compounds. All components are related to improving lipid profiles and glycemic control, reducing blood pressure and inflammation. Regular intake of a moderate mix of nuts and legumes can optimize these benefits. Importantly, these effects are achieved without promoting weight gain [30]. Moreover, observational studies consistently show that higher nuts consumption is associated with reduced risk of CVDs, coronary heart disease (CHD), and total mortality. A daily intake of around 15–20 g of nuts offers optimal protection, with little additional benefit beyond this amount. Moreover, whole nuts (especially walnuts and peanuts) are more effective than processed products like peanut butter. Although some uncertainties remain, especially regarding stroke subtypes, heart failure, and peripheral artery disease, regular nut consumption is strongly recommended for CVD prevention [31].

The macronutrient composition of nuts (Table 1), which are nutrient‐dense and rich in carbohydrates, proteins, and fats, contributes 550–720 kcal per 100 g. Nuts are among the highest‐fat PBD, although their fat profile is healthy, with low saturated fatty acids (5%–15%) and high unsaturated fatty acids (MUFAs and PUFAs). Walnuts are particularly rich in linoleic acid, α‐linolenic acid (Omega‐3), and PUFAs, while nuts such as hazelnuts, almonds, pistachios, peanuts, and cashews contain more MUFAs [32]. Their micronutrient contents (Table 2) include iron, magnesium, zinc, selenium, and vitamins B2 and B6; key factors in one‐carbon metabolism related to DNA methylation; and vitamin E. Each nut has unique benefits: hazelnuts and walnuts offer antioxidants and minerals, almonds and cashews are nutrient‐dense, peanuts are high in vitamin E and folate, and pistachios provide vitamin B6. A variety of nuts is recommended to ensure a balanced intake of essential nutrients and antioxidants [28].

**TABLE 1:** Average macronutrient composition of nuts (per 100 g).

Nuts	Energy (kcal)	Carb (g)	Protein (g)	Fat (g)	Fiber (g)	SFA (g)	MUFA (g)	PUFA (g)	LA (g)	ALA (g)
Walnuts	619	9.6	24.1	59.3	6.8	3.5	15.4	36.4	33.8	2.680
Hazelnuts	628	16.7	15.0	60.8	9.7	4.5	45.7	7.9	7.8	0.087
Almonds	579	21.6	21.2	49.9	12.5	3.8	31.6	12.3	12.3	0.003
Pistachios	560	27.2	20.2	45.3	10.6	5.9	23.3	14.4	14.1	0.289
Peanuts	587	21.3	24.4	49.7	8.4	7.7	26.2	9.8	9.7	0.025
Macadamias	718	13.8	7.9	75.8	8.6	12.1	58.9	1.5	1.3	0.206
Cashews	553	30.2	18.2	43.8	3.3	7.8	23.8	7.8	7.8	0.062

Abbreviations: ALA, α‐linolenic acid (Omega‐3); LA, linoleic acid (Omega‐6); MUFA, monounsaturated fatty acids; PUFA, polyunsaturated fatty acids; SFA, saturated fatty acids [32].

**TABLE 2:** Average micronutrient composition of nuts (per 100 g).

Nuts	Fe (mg)	Mg (mg)	Zn (mg)	Cu (mg)	Se (µg)	Vit B2 (mg)	Vit B6 (mg)	Folate (µg)	Vit E (mg)
Walnuts	3.12	201	3.37	1.36	17	0.13	0.58	31	2.1
Hazelnuts	4.70	163	2.45	1.72	2.4	0.11	0.56	113	15.0
Almonds	3.71	270	3.12	1.03	4.1	1.14	0.14	44	25.6
Pistachios	3.92	121	2.20	1.30	7.0	0.16	1.70	51	2.9
Peanuts	1.58	178	2.77	0.43	9.3	0.20	0.47	97	4.9
Macadamias	3.69	130	1.30	0.76	3.6	0.16	0.27	11	0.5
Cashews	6.68	292	5.78	2.20	19.9	0.06	0.42	25	0.9

Abbreviations: Cu, copper; Fe, iron; Mg, magnesium; Se, selenium; Zn, zinc; Vit B2, Riboflavin; Vit B6, Pyridoxine; Vit E, Vitamin E (alpha‐tocopherol) [32].

Next, the macronutrient composition of legumes (Table 3), known for their high protein and essential mineral content, including calcium and iron, makes them a key energy source [33]. Legumes typically contain approximately 60 g of carbohydrates and 20 g of protein per 100 g, making them one of the richest plant‐based sources of these macronutrients. Soybeans are the most energy‐dense, providing 446 kcal and 36.5 g of protein per 100 g, along with 19.9 g of fat and the highest PUFA content (11.3 g), mainly from linoleic and alpha‐linolenic acids. Chickpeas and mung beans are carbohydrate‐rich (63.0 g and 62.6 g per 100 g) and provide sustained energy. Chickpeas also contain 6.0 g of fat, with a good balance of MUFAs (1.38 g) and PUFAs (2.73 g). Green peas lead in fiber (22.2 g per 100 g), followed by mung beans (16.3 g), red beans (15.2 g), and black beans (15.5 g), supporting digestive health and blood sugar management. Saturated fats in most legumes are low, with soybeans containing 2.88 g per 100 g, balanced by their high unsaturated fat content, which supports heart health [32]. Overall, the choice of legumes can depend on dietary goals. Soybeans are ideal for high protein and fat intake, while green peas and mung beans offer high fiber and moderate protein. Chickpeas provide a balanced macronutrient profile, making them versatile. Red and black beans are great for moderate energy, with a focus on fiber and protein. Each legume's unique benefits make it valuable in a varied, balanced diet.

**TABLE 3:** Average macronutrient composition of legumes (per 100 g).

Legumes	Energy (kcal)	Carb (g)	Protein (g)	Fat (g)	Fiber (g)	SFA (g)	MUFA (g)	PUFA (g)	LA (g)	ALA (g)
Red beans	337	61.3	22.5	1.1	15.2	0.15	0.08	0.59	0.23	0.36
Green peas	364	61.6	23.1	3.9	22.2	0.41	0.62	1.02	0.86	0.16
Chickpeas	378	63.0	20.5	6.0	12.2	0.60	1.38	2.73	2.63	0.10
Mung beans	347	62.6	23.9	1.2	16.3	0.35	0.16	0.38	0.36	0.02
Soybeans	446	30.2	36.5	19.9	9.3	2.88	4.40	11.3	9.92	1.33
Black beans	341	62.4	21.6	1.4	15.5	0.37	0.12	0.61	0.33	0.28

Abbreviations: ALA, α‐linolenic acid; LA, linoleic acid; MUFA, monounsaturated fatty acids; PUFA, polyunsaturated fatty acids; SFA, saturated fatty acids [32].

The nutritional composition of the various legumes revealed significant differences in their mineral and vitamin contents (Table 4), making them essential components of a balanced diet. Among the legumes listed, soybeans stand out as the most nutrient‐dense option, providing the highest levels of iron (15.7 mg), magnesium (280 mg), zinc (4.89 mg), copper (1.66 mg), selenium (17.8 µg), and vitamin B2 (0.87 mg). They also offer a substantial amount of vitamin B6 (0.38 mg) and vitamin E (0.85 mg), although their folate content (375 µg) is slightly lower than that of chickpeas and mung beans [32]. The data highlight the diverse nutritional profiles of legumes, emphasizing their role as excellent plant‐based sources of essential minerals, vitamins, and antioxidants. Soybeans and mung beans are particularly nutrient‐dense, while chickpeas and black beans offer unique benefits in terms of specific nutrients. Including a variety of legumes in the diet can help ensure numerous nutritional benefits.

**TABLE 4:** Average micronutrient composition of legumes (per 100 g).

Legumes	Fe (mg)	Mg (mg)	Zn (mg)	Cu (mg)	Se (µg)	Vit B2 (mg)	Vit B6 (mg)	Folate (µg)	Vit E (mg)
Red beans	6.69	138	2.79	0.70	3.2	0.22	0.40	394	0.21
Green peas	4.73	63	3.49	0.81	10.7	0.24	0.14	15	0.12
Chickpeas	4.31	79	2.76	0.66	0	0.21	0.53	557	0.82
Mung beans	6.74	189	2.68	0.94	8.2	0.23	0.38	625	0.51
Soybeans	15.7	280	4.89	1.66	17.8	0.87	0.38	375	0.85
Black beans	5.02	171	3.65	0.84	3.2	0.19	0.29	444	0.21

Abbreviations: Cu, copper; Fe, iron; Mg, magnesium; Se, selenium; Zn, zinc; Vit B2, Riboflavin; Vit B6, Pyridoxine; Vit E, Vitamin E (alpha‐tocopherol) [32].

### 3.2 Phytochemicals in Nuts and Legumes

Phytochemicals are active compounds in plants that offer various health benefits. Nuts and legumes are especially abundant in these compounds, enhancing their nutritional and medicinal properties. The following summarizes the key phytochemicals found in nuts and legumes (Table 5).

**TABLE 5:** Key phytochemicals in nuts and legumes: molecular weight, chemical structures, and antiplatelet mechanisms.

Compound name	Sources	Phytochemical class	Molecular weight (g/mol)	Chemical structure	Reference
Ellagic acid	Walnut	Polyphenol	302.19	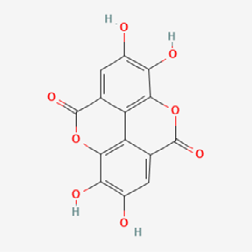 C_14_H_6_O_8_	[34]
Catechin	Peanut	Flavonoids	290.27	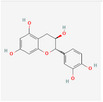 C_15_H_14_O_6_	[35]
Quercetin	Almond, pistachio	Flavonoids	302.24	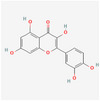 C_15_H_10_O_7_	[36]
Genistein	Soybean	Flavonoids	270.24	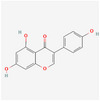 C_15_H_10_O_5_	[37]
Ferulic acid	Almond	Phenolic acid	194.18	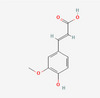 C_10_H_10_O_4_	[38]
Gallic acid	Chickpea	Phenolic acid	170.12	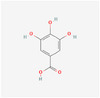 C_7_H_6_O_5_	[39]
Caffeic acid	Almonds Walnuts Cashews	Phenolic acid	180.16	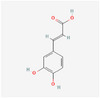 C_9_H_8_O_4_	[40]

#### 3.2.1 Polyphenol

Polyphenols, a diverse group of phytochemicals with potent antioxidant properties, play a key role in reducing oxidative stress and inflammation, which are linked to chronic diseases such as CVDs, diabetes, and cancer. Polyphenols also contribute to gut health by modulating gut microbiota, leading to systemic health benefits. Nuts and legumes, such as walnuts, almonds, pecans, and peanuts, are particularly rich sources of polyphenols, including flavonoids, tannins, lignans, and phenolic acids [41, 42]. Specific examples include ellagic acid in walnuts, which can modulate platelet activity through its antioxidant properties, and catechins in peanuts, which inhibit PA by enhancing NO signaling and reducing ROS production [41, 43]. Almonds contain phenolic compounds such as quercetin and isorhamnetin [44], which are known to exhibit anti‐inflammatory and antithrombotic effects, while pecans are rich in proanthocyanidins, which also show potential in reducing platelet activation [45, 46]. The proposed antiplatelet pathways for polyphenols include inhibition of cyclooxygenase (COX) activity, suppression of thromboxane A2 production, and modulation of platelet signaling pathways, leading to decreased platelet activation and PA [43, 47]. These findings highlight the significant role of polyphenols in supporting cardiovascular health and preventing platelet‐mediated disorders.

##### 3.2.1.1 Flavonoid

Flavonoids, a subgroup of polyphenols, have been extensively studied for their diverse health benefits, including anti‐inflammatory, anticancer, and cardioprotective effects [48]. These compounds improve endothelial function, reduce blood pressure, and markedly promote cardiovascular health. Nuts such as pistachios, cashews, and hazelnuts, as well as legumes like black beans, kidney beans, and soybeans, are excellent sources of flavonoids, including genistein, daidzein, and quercetin [49, 50]. Quercetin in pistachios inhibits PA by blocking glycoprotein IIb/IIIa activity, a key receptor in platelet adhesion [51]. Similarly, soybean genistein reduces platelet activation by suppressing the tyrosine kinase pathways essential for platelet signaling. Flavonoids also modulate endothelial nitric oxide synthase (eNOS) activity, enhancing NO production and promoting vasodilation. Furthermore, these compounds inhibit platelet activation by targeting adhesion receptors and suppressing pathways mediated by ADP and thrombin receptors, thereby reducing thrombotic risk [50]. These findings highlight the critical role of flavonoids in supporting cardiovascular health and preventing platelet‐mediated disorders.

##### 3.2.1.2 Phenolic Acid

Phenolic acids, a class of phytochemicals with potent antioxidants and anti‐inflammatory properties, play a vital role in protecting cells from oxidative damage and in reducing the risk of chronic diseases [52]. They also exhibit antimicrobial effects, contributing to food preservation and gut health. Nuts like almonds and hazelnuts are rich sources of phenolic acids such as caffeic acid and ferulic acid, while legumes such as lentils and chickpeas contain compounds like p‐coumaric acid and gallic acid [53, 54]. Ferulic acid in almonds inhibits PA by suppressing COX‐1 and COX‐2 enzyme activity and reducing thromboxane A2 production [55]. Similarly, gallic acid in chickpeas decreases thromboxane A2 synthesis and inhibits calcium mobilization in platelets, further lowering their activation potential [54]. Phenolic acids achieve their antiplatelet effects by modulating cyclooxygenase activity and disrupting calcium signaling pathways, making them valuable compounds for cardiovascular health [53].

#### 3.2.2 Polyphenol–Protein Complexes

Polyphenol–protein complexes are formed when polyphenols bind with proteins during food processing or digestion, influencing the bioavailability and functionality of both components and amplifying their health benefits. These complexes also impact food texture, stability, and flavor, making them significant in food science and product development. Such interactions are commonly observed in nuts like almonds and walnuts and legumes like soybeans and lentils [56]. Polyphenol–protein complexes form through covalent and noncovalent interactions, markedly enhancing the stability, bioavailability, and bioactivity of polyphenols. Covalent interactions, such as enzymatic crosslinking or oxidative coupling, create stable bonds that protect polyphenols from degradation, whereas noncovalent forces, including hydrogen bonding, hydrophobic interactions, and ionic binding, contribute to reversible complex formation. These complexes improve polyphenol solubility, protect them from gastrointestinal breakdown, and facilitate controlled release in the target tissues. In addition, they enhance functional properties such as emulsification, gelation, and antioxidant activity, making them valuable in nutraceuticals and food applications [57]. For example, EGCG‐ovalbumin, resveratrol‐β‐lactoglobulin, and curcuminzein complexes exhibit improved therapeutic efficacy, particularly in conditions such as ulcerative colitis, diabetes, and CVDs [57]. According to Jiang et al. [58], the study demonstrated that non‐covalent complexes between chlorogenic acid and milk proteins, both whey protein isolate and casein, improved protein digestibility by partially unfolding protein structures, making them more accessible to digestive enzymes. The resulting peptides and chlorogenic acid showed synergistic antioxidant activity, enhancing their potential for oxidative stress, improving protein solubility and foaming properties, suggesting potential applications in functional food products, and may contribute to better nutritional and health benefits through enhanced bioavailability and antioxidant defense [58]. Moreover, Xiong et al. [59] demonstrated that polyphenol–protein complexes significantly enhance the stability and bioactivity of berry pomace polyphenols during digestion by forming aggregates with rice and pea proteins. These complexes protect polyphenols from degradation and allow more to reach the colon for microbial metabolism. This leads to enhanced antioxidant and anti‐inflammatory effects [59]. Additionally, Hoskin et al. [60] studied polyphenol–protein complexes derived from blueberry pomace. The results demonstrated multiple health‐related bioactivities by reducing oxidative stress by inhibiting ROS and NO production and suppressing inflammation by downregulating pro‐inflammatory genes such as *COX‐2*, *IL‐1β*, and *iNOS* [60]. These complexes stabilize polyphenols, prolong their bioactivity, and enhance their ability to suppress platelet activation and PA. They may also interfere with fibrinogen binding to platelet receptors, minimizing the risk of clot formation [56].

## 4 Nuts and Legumes Related to PA Through DNA Methylation

### 4.1 The Effect of Nuts and Legumes on Anti‐PA/CVD

The consumption of nuts and legumes has been associated with potential cardiovascular benefits, partially due to the effects on PA and vascular health, as supported by several studies (Table 6). Nuts and legumes contain key components that may contribute to reduced oxidative stress, improved lipid profiles, and modulation of platelet function. For example, Baru almond oil is rich in tocopherols and unsaturated fatty acids, which have been shown to reduce oxidative stress and platelet hyperreactivity, potentially supporting endothelial function in male Wistar rats [61]. Walnut bark extract contain bioactive compounds such as rutin, gallic acid, and flavonoids, which may inhibit platelet activation and PA while reducing thromboxane formation, studied on in vitro experiments using human platelet‐rich plasma (PRP) [62]. Hazelnuts provide MUFAs and PUFAs, vitamin E, fiber, and antioxidants that lower LDL cholesterol and improve vascular health, as reported in human clinical trials involving hypercholesterolemic conditions [63]. Peanuts contain oleic acid, resveratrol, and phytosterols, which may help reduce cholesterol and PA, with supporting evidence from the RCT in obese women and the systematic review and meta‐analysis of RCT showing improved body composition, reductions in LDL cholesterol, platelet count, and homocysteine levels following whole peanut consumption within an energy‐restricted diet [64, 65]. Among legumes, the common bean (*Phaseolus vulgaris*) are rich in proteins, phenols, and flavonoids, which are related to reducing oxidative stress and PA, in vitro models by using human PRP [66]. Chickpeas provide fiber, polyphenols, saponins, and vitamins C and E, which may support lipid regulation and cardiovascular protection, supported by in vitro findings in human blood samples [67]. Lastly, soybeans are abundant in isoflavones and phytoestrogens, which are associated with decreased PA and oxidative stress while promoting cardiovascular health, based on evidence from randomized crossover trials in postmenopausal women [68]. Data from studies demonstrate a positive association between nut and legume consumption and improvements in cardiometabolic markers. The studies use various methodologies from in vitro mechanistic assays to RCTs in at‐risk populations, presenting both mechanistic and clinical understandings. However, several limitations persist. The human studies focus on specific groups, such as only women, particularly postmenopausal or obese individuals, and sample sizes were often small (*n* < 30). Notably, in vitro and in vivo studies provided mechanistic hypotheses involving AKT pathways, TXA2 receptor modulation, and oxidative stress reduction, yet the translational value to human clinical settings remains uncertain. Another important consideration is the heterogeneity in the type and cultivated variety of nuts and legumes used across studies. Most interventions focus on a single form of a nut or legume (e.g., *P. vulgaris*, *Cicer arietinum*, or *Arachis hypogaea*), without examining whether the observed effects are consistent across different varieties or processing methods. For example, the composition of bioactive compounds such as polyphenols, phytosterols, tocopherols, or nutrients such as fatty acid profiles can vary significantly between cultivars, growing conditions, and even post‐harvest processing. This variation may influence biological activity, including effects on platelet function, oxidative stress, and lipid metabolism. While current data suggest profitable cardioprotective effects of nuts and legumes, larger, long‐term RCTs with diverse populations and standardized endpoints are needed to confirm causality and elucidate underlying mechanisms. Despite the consistency in reported improvements in surrogate markers such as LDL cholesterol, platelet activity, and body composition, current evidence remains associative mainly. Most studies do not adequately account for confounding variables, and the designs, particularly small‐scale or short‐duration trials, limit the ability to assume a direct causal relationship between nut or legume consumption and cardiometabolic health outcomes.

**TABLE 6:** Nuts and legumes consumption and their impact on CVD via PA.

Type	Key component	Effect of PA or CVD outcomes
**Nuts**		
Almonds [61]	Tocopherols (α, β + γ, δ)	↓ Oxidative stress → ↓ PA
	Antioxidant compounds	↓ ROS → ↓ platelet hyperreactivity and ↑ antithrombotic effect
	Unsaturated fatty acids	Improve lipid profile, ↓ CVD risk (anti‐inflammatory)
	Oleic acid	Anti‐inflammatory → ↑ endothelial function, ↓ thrombosis
	Linoleic acid and Linolenic acid	↑ Antioxidant activity → ↑ vascular health
Walnuts [62]	Rutin	Inhibit PA via blocking glycoprotein IIb/IIIa and ↑ nitric oxide
	Gallic acid	Antioxidant/anti‐inflammatory → ↓ platelet adhesion
	Flavonoids	↓ Thromboxane, ↑ cAMP/cGMP → ↓ PA
	Coumarins	↓ Phospholipase C → ↓ platelet activation
	Phenolic compounds	↓ Oxidative stress and inflammation → ↓ thrombotic events
Hazelnut [63]	Monounsaturated fatty acids (MUFA)	↓ LDL and total cholesterol → ↓ CVD risk
	Polyunsaturated fatty acids (PUFA)	↓ Triglycerides, improve LDL particle size
	Vitamin E (α‐tocopherol)	↓ LDL oxidation, ↑ vascular health
	Dietary fiber	↓ Total cholesterol and LDL
	L‐arginine	↑ Nitric oxide → ↑endothelial function
	Folic acid	Support endothelial function (no significant homocysteine effect)
	Phytosterols	Compete with cholesterol → ↓ absorption
	Antioxidants	↓ Oxidative stress and inflammation
Peanut [64, 65]	Monounsaturated fatty acids (MUFA)	↓ LDL, improve lipid profile, ↓ platelet aggregation
	Polyunsaturated fatty acids (PUFA)	↓ Triglycerides and LDL
	Resveratrol (skin)	Antioxidant → ↓ platelet aggregation
	Phytosterols	↓ Cholesterol absorption
	Dietary fiber	↓ Cholesterol → ↑cardiovascular outcome
	Vitamin E	↓ LDL oxidation, ↑ vascular health
	Oleic acid	↓ Blood pressure and platelet aggregation
	Catechins and procyanidins (skin)	Strong antioxidant/anti‐inflammatory → ↓ oxidative stress
**Legumes**		
Beans (*Phaseolus vulgaris*) [66]	Proteins	Modulate platelets, support vascular health
	Total phenols	↓ Oxidative stress and platelet aggregation
	Dietary fiber	Improve lipid profile, ↓ inflammation
	Flavonoids (e.g., Nobiletin)	↓ Platelet aggregation, ↓ P‐selectin
	Antioxidants	Free‐radical scavenging → prevent oxidative damage
Chickpeas [67]	Proteins	Regulate platelet function, support heart health
	Dietary fiber	↓ Cholesterol, improve gut and cardiovascular health
	Polyphenols	↓ PA and oxidative stress
	Saponins	↓ Cholesterol and inflammation
	Fatty acids (oleic and linoleic)	Improve lipid profile, ↓ CVD risk
	Vitamin C and E	Antioxidants → inhibit platelet aggregation
	Phenolic acids	↓ PA and blood pressure
	Tannins	Anti‐inflammatory/antioxidant → inhibit platelet activation
Soybeans [68]	Isoflavones (Genistein and Daidzein)	↓ Thromboxane A2 receptor density → ↓ platelet aggregation
	Polyphenols	↓ Oxidative stress and inflammation
	Phytoestrogens	Mimic estrogen → protect cardiovascular system

### 4.2 DNA‐Methylation

Epigenetics is the study of heritable changes in gene expression that occur without modifying the underlying DNA sequence. It plays a crucial role in regulating complex biological processes, including those related to cardiovascular diseases (CVDs). DNA methylation is a biological process in which a methyl group (CH_3_) is added to a DNA strand, specifically at the 5‐carbon position of a cytosine ring. This modification is catalyzed by enzymes known as DNA methyltransferases, including DNMT1, DNMT3a, and DNMT3b. Methylation typically occurs in regions that contain a high frequency of cytosine‐guanine dinucleotides (CpGs) [69]. DNMT1 is responsible for maintaining DNA methylation, while de novo methylation is carried out by the enzymes DNMT3a and DNMT3b. Methylation in the promoter region of genes leads to the suppression of gene expression. This can occur through several mechanisms, such as hindering the accessibility of transcription factors and activators. Changes in chromatin structure resulting from this process can prevent transcription from occurring. Additionally, the recruitment of repressor proteins to the promoter region is another important strategy for gene expression regulation [70].

#### 4.2.1 DNA‐Methylation Related to PA

DNA methylation influences genes that play a role in platelet aggregation. Specifically, the patterns of DNA methylation can enhance or suppress the expression of genes related to platelet activation, adhesion, and aggregation, ultimately affecting the entire process of blood clot formation.

According to Yamada et al.’s study in 2014, the hypomethylation of nucleotide‐binding oligomerization domain‐containing 2 (NOD2) and early B‐cell factor 1 suggests increased inflammatory signaling, which can enhance platelet activation and PA. Conversely, hypermethylation of the FYN oncogene related to SRC, FGR, and YES (FYN) may impair platelet function, altering the thrombotic potential in atherosclerotic lesions. Thus, these methylation changes contribute to a proinflammatory and thrombogenic environment, influencing PA and atherosclerosis progression [71]. Yang et al., in 2023, explored the impact of both DNA methylation and genetic variation of the platelet endothelial aggregation receptor 1 (*PEAR1*) gene (rs12041331) on platelet reactivity in patients with recurrent ischemic stroke treated with clopidogrel. PEAR1, a transmembrane protein found in platelets and endothelial cells, is essential for platelet activation and PA via integrin αIIbβ3 activation [72]. Genetic variations, such as single‐nucleotide polymorphisms, in the *PEAR1* gene are linked to increase PA and may influence the effectiveness of antiplatelet therapies and CVD risk. Some PEAR1 variants also impact endothelial function, potentially affecting vascular health and contributing to CVD progression [73]. The results showed that the major G allele of the PEAR1 rs12041331 was associated with hypermethylation at the *PEAR1* gene locus. Hypermethylation of PEAR1 correlated with higher mRNA expression of the gene in platelets. This higher expression of PEAR1 was related to high on‐treatment platelet reactivity, meaning that platelets remained overly active despite clopidogrel treatment. In contrast, the A allele was associated with lower methylation and platelet reactivity, but its role in recurrent stroke remains uncertain [74]. In addition, mitochondrial genes *MT‐CO1* and *MT‐CO3* encode key subunits of cytochrome c oxidase, the enzyme responsible for the final step in the mitochondrial electron transport chain that facilitates ATP synthesis [75, 76]. The MT‐CO1 and MT‐CO3 subunits are vital for the enzyme's catalytic activity, contributing to electron transfer and proton translocation [77, 78]. Changes in the function of these mitochondrial genes can markedly affect cardiovascular health. Altered methylation of MT‐CO1 and MT‐CO3 has been observed in patients with CVD, potentially disrupting mitochondrial function and promoting abnormal platelet behavior, contributing to CVD development [73]. According to Peng et al., further investigation of mitochondrial DNA (mtDNA) methylation levels in platelets and their association with MI. The results reported that MT‐COX1 and MT‐COX3 hypermethylation may reduce mitochondrial function, leading to higher ROS production and oxidative stress. Increased ROS triggers platelet activation, leading to higher platelet reactivity and atherothrombosis, which are major contributors to MI. Meanwhile, hypomethylation of MT‐ATP6 may increase gene expression to compensate for the high energy demand during platelet activation. This may lead to increased ATP synthesis, enhancing platelet activation and PA, and further contributing to thrombus formation [79].

### 4.3 Dietary Contributions to DNA Methylation

**FIGURE 3:**
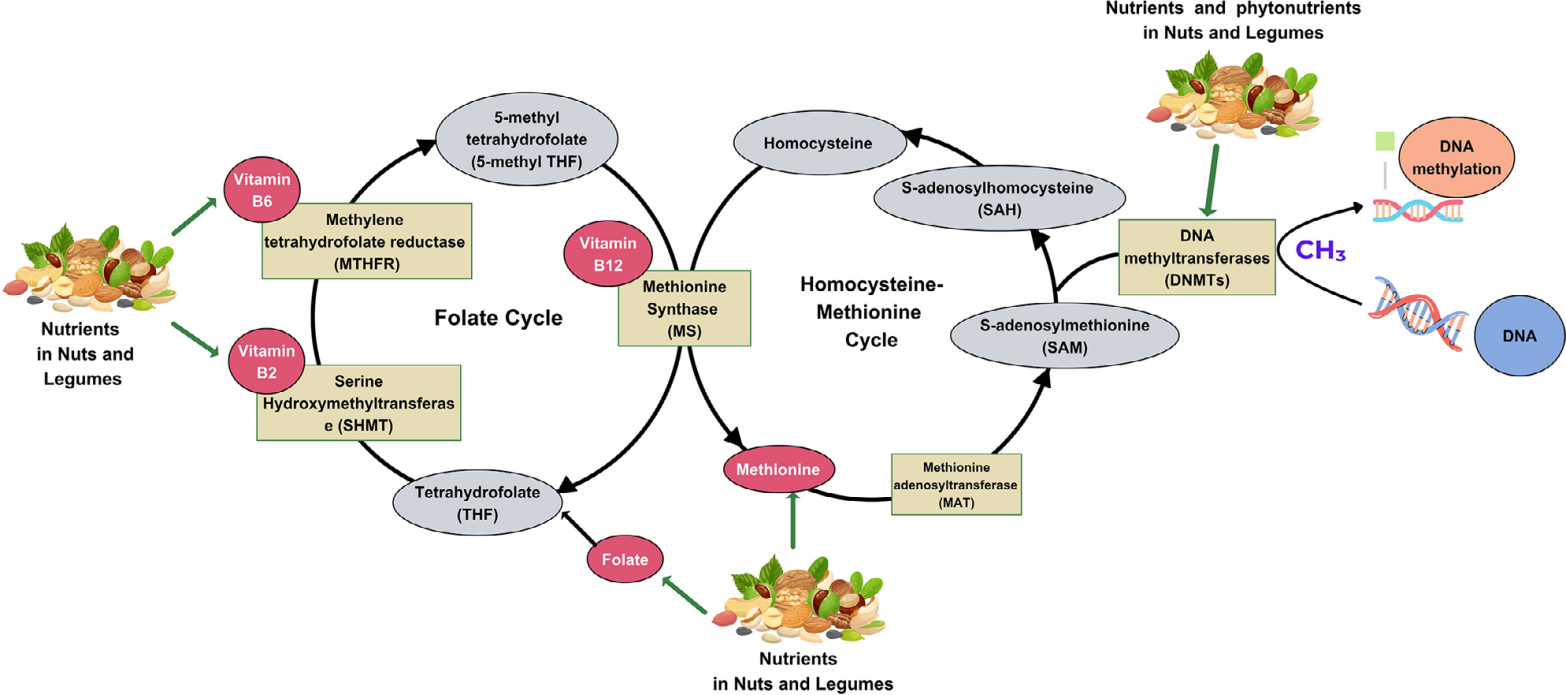
The interaction between diet (nuts and legumes) and DNA methylation related to folate cycle and the homocysteine‐methionine cycle (created with CAVA.com, by Siwaphorn Chaimati).

Nuts and legumes are rich sources of essential nutrients for modulating folate cycle and homocysteine‐methionine cycle related to DNA methylation mechanism, such as folate, vitamin B12, methionine, and choline, which are integral to the one‐carbon metabolism pathway (Figure 3). This pathway produces S‐adenosylmethionine (SAM), a universal methyl donor essential for DNA methylation. Adequate levels of these nutrients’ intake help maintain normal methylation patterns and preventing aberrant gene expression associated with platelet hyperactivity. Folate, often abundant in legumes, plays a particularly critical role as a carrier of the methyl groups necessary for DNA methylation [80].

Dietary interventions, involving nuts and legumes, are associated with modifications in DNA methylation patterns. These epigenetic modifications are increasingly explored as potential mechanisms through which diet may influence health‐related pathways, including those relevant to PA and CVD risk. However, direct causal relationships have not been verified [81, 82, 83]. Table 7 summarizes studies on the effect of nuts and legume consumption on DNA methylation. A study by Lima et al. involved 40 overweight or obese women in a double‐blind, placebo‐controlled intervention that assessed the effects of folate and hazelnut oil intake on *ADRB3* gene methylation, revealing that dietary fats may be related to obesity‐related epigenetics [84]. Gensous et al. studied older adults in a 1‐year dietary intervention (NU‐AGE) and found that a Mediterranean diet rich in nuts and legumes was associated with methylation profiles suggestive of epigenetic rejuvenation, particularly among individuals with a higher baseline epigenetic age. Although promising, the long‐term health implications of such changes remain to be clarified [85]. Kim et al. analyzed 1995 participants from the Framingham Heart Study and showed that higher overall diet quality, including increased nut and legume consumption, was associated with slowed epigenetic aging through methylation changes in aging‐related markers like GrimAge and PhenoAge [86]. Similarly, Arpón et al. assessed participants from the PREDIMED‐Navarra trial over 5 years, reporting that Mediterranean diets enriched with extra‐virgin olive oil or nuts were associated with DNA methylation changes in genes involved in metabolic, inflammatory, and diabetes‐related pathways. These findings support a possible role for dietary fat quality in modulating gene regulation, although the specific clinical implications remain uncertain [87]. Finally, a meta‐analysis of epigenome‐wide association studies (EWAS) on the relationship between food intake and DNA methylation by Hellbach et al. in 2023 included 2315 middle‐aged to older adults from the following three cohorts: KORA (Germany); LLS (Netherlands); and TwinsUK (UK, female‐only). The study found significant associations between the consumption of high‐fat food groups such as nuts and seeds, cream, butter, and alcoholic beverages and changes in CpG methylation. Nut and seed consumption were significantly associated with changes in DNA methylation at 18 CpG sites. These methylation changes were linked to metabolic regulation and energy homeostasis genes, suggesting potential epigenetic mechanisms through which nuts and seeds may influence health. Notably, affected genes included *GLI1*, which regulates fat accumulation through the Hedgehog signaling pathway. *ATP5H*—related to mitochondrial energy production. *NTN1*, *PIP4K2C*, *WEE1*, and *SYT1* are associated with cell signaling and metabolism. These findings indicate that nuts and seeds may impact cardiometabolic health through nutrient content and epigenetic modulation, reinforcing their role in health‐promoting dietary patterns [88].

**TABLE 7:** Summary of studies on the impact of nuts and legume consumption on DNA methylation.

Authors	Population	Study design	Methylation Assay	Conclusions/ Outcomes
Lima et al. (2017) [84]	40 overweight/obese women (BMI 25–35, age 20–59)	RCT (four groups; folate and hazelnut oil; 8 wks)	Pyrosequencing (*ADRB3* gene)	‐ Hazelnut oil→ ↓ ADRB3 methylation. ‐ High folate→ ↑ methylation. ‐ Low‐folate + hazelnut oil group → the greatest methylation reduction and improved LDL‐C, waist circumference, and fat intake.
Gensous et al. (2020) [85]	Older adults (Italy and Poland)	One‐year dietary intervention study (NU‐AGE project)	Illumina HumanMethylation 450 BeadChip	‐ MedDiet (rich in nuts and legumes) → methylation changes in inflammation, immunity, metabolism genes ‐ Promoted epigenetic rejuvenation, especially in participants with higher baseline epigenetic age.
Kim et al. (2022) [86]	1995 participants from the Framingham Heart Study	Observational study (diet quality via DASH score)	Illumina HumanMethylation450 BeadChip	‐ Higher diet quality (nuts/legumes/beans) → slower epigenetic aging. ‐ Methylation changes in aging markers (GrimAge, PhenoAge, DunedinPoAm) → related to longevity and metabolic health
Arpón et al. (2022) [87]	36 older adults (60–70 years) from PREDIMED‐Navarra	RCT (three groups: MedDiet + EVOO, MedDiet + Nuts, low‐fat control), followed for 5 years	Infinium HumanMethylation450K BeadChip	‐ MedDiet + Nuts → ↑ CPT1B methylation (fat metabolism) ‐ MedDiet + EVOO → ↓ GNAS methylation (glucose regulation) ‐ PUFA intake correlated with these changes → highlights fat quality's role in epigenetic regulation
Hellbach et al. (2023) [88]	2315 adults from three cohorts: ‐ KORA (Germany) ‐ LLS (the Netherlands) ‐ TwinsUK (females UK)	EWAS meta‐analysis	‐ KORA: EPIC BeadChip ‐ LLS & TwinsUK: 450K BeadChip	‐ DNA methylation linked to intake of high‐fat foods (e.g., onions/garlic, nuts/seeds, dairy, plant oils, alcohol) ‐ CpG targets: GLI1, ATP5H, MYC, RPTOR, ASAM, FOXA2, DIO1, CLIP2, CYFIP1, PHGDH, TRA2B.

Despite increasing interest in the potential function of nut and legume consumption in modulating DNA methylation and their implications for cardiometabolic health, several limitations of previous studies should be recognized when interpreting the current evidence. Most existing studies report associations between dietary patterns and DNA methylation changes, but do not specify causality because observational studies are limited by potential confounding factors such as dietary factors, overall diet quality, physical activity, genetic background, and socioeconomic status. Although RCTs are considered, many focus on short‐term dietary interventions, involve small sample sizes, and rely on biomarkers, such as selected CpG methylation sites, rather than tracking long‐term clinical outcomes. Moreover, substantial heterogeneity in types, doses, and preparation methods of nuts and legumes studied makes it challenging to determine which components are responsible for the observed effects. Most findings do not confirm that methylation changes lead to functional gene expression and physiological impact. Moreover, the generalizability of current results is relied on the homogenous study populations, which are usually limited to specific age groups, sexes, or ethnic backgrounds. So, the effects of diet on epigenetic markers may vary across demographic groups due to differences in genetics, lifestyle, and baseline dietary patterns. While observational studies provide establish correlations, and well‐designed RCTs have the potential to infer causality, the various limitations discussed, such as small sample sizes, short intervention durations, and reliance on surrogate endpoints, suggest that current evidence provides only limited support for causal relationships.

### 4.4 Effect of Bioactive Compounds in Nuts and Legumes on DNA Methylation Related to PA

Beyond nutrients, nuts and legumes are rich in bioactive compounds such as flavonoids and polyphenols, which may exhibit epigenetic regulatory properties [89, 90]. Studies directly exploring the mechanisms for inhibiting DNMT through the consumption of nuts and legumes remain unavailable. However, studies have shown that phenolic compounds in green tea, such as epigallocatechin gallate (EGCG), are related to inhibiting DNMT activity. Although no direct studies have been conducted on DNMT inhibition by nuts or legumes, the consumption of foods rich in phenolic compounds and methyl‐donating nutrients may influence DNMT activity and DNA methylation. These compounds are related to modulating DNMT activity and potentially influence PA pathways [91]. DNA methylation plays a pivotal role in platelet function by modulating the expression of genes involved in platelet activation and PA [92]. Many studies have highlighted the ability of bioactive compounds include polyphenols, flavonoids, phenolic acids, and isoflavones in nuts and legumes to influence these epigenetic processes, and interact with DNMTs and provide the methyl donors necessary for normal methylation processes, offering significant cardiovascular health benefits [93].

As listed in Table 8, for example, catechins in peanuts, soybeans, and pistachios exhibit roles in epigenetic regulation and cardiovascular protection. In vitro studies using SssI DNMT and human DNMT1 have shown that catechin, epicatechin, and EGCG modulate DNMT‐mediated methylation, with EGCG acting as a potent DNMT inhibitor enhanced by Mg^2^⁺ [94], and CpG demethylation and reactivation of silenced genes such as *p16* and *RARβ* [95]. Furthermore, green tea catechins suppress platelet aggregation (PA) via Ca^2^⁺/IP_3_ signaling pathway, indicating antithrombotic potential [96]. Quercetin in almonds, pistachios, and walnuts downregulates DNMTs and STAT3, demethylates pro‐apoptotic genes (*BCL2L11*, *DAPK1*), and enhances histone acetylation in leukemia and xenograft models [97], while protecting against nickel‐induced liver injury via Nrf2/HO‐1 activation [98]. It also inhibits platelet activation by binding GPIIb/IIIa and reducing PDMP release [99]. Caffeic acid and its derivative Caffeic Acid Phenethyl Ester (CAPE), found in almonds, walnuts, and cashews, show the function of inhibiting DNMT1 activity and reducing DNA methylation in both in vitro and animal studies, modulating genes such as the agouti locus [100]. Caffeic and chlorogenic acids inhibit DNMT via COMT‐mediated SAH formation in vitro, enzymatic assay, and cell culture with breast cancer cells (MCF‐7, MDA‐MB‐231) [101]. Moreover, suppressing thrombin‐induced platelet activation through cAMP elevation and ERK/Akt inhibition [102]. Next is gallic acid, present in walnuts, hazelnuts, and pecans, which decreases DNMT1, DNMT3A, and DNMT3B levels, induces demethylation, and reactivates GADD45, enhancing chemosensitivity to cisplatin [103], while simultaneously binding to thrombin residues to reduce platelet activation and protect endothelial cells from apoptosis [104]. Finally, the soy isoflavones genistein and daidzein consistently demonstrate DNA demethylation via DNMT1 inhibition in an in vitro study on MCF‐7 and MDA‐MB‐231 cells [105], in clinical studies of the RCT trial on prostate cancer patients showing modulation of MYC, PTEN, and EMT‐related pathways [106]. However, soy isoflavones may inhibit DNMTs and HDACs, short‐term intake showed no significant methylation changes [107]. These compounds are also related to cardioprotective effects by inhibiting platelet aggregation, reducing oxidative stress by activating Nrf2/HO‐1 pathway [108], and reducing NO and TNF‐α production in macrophages lower MCP‐1 levels in endothelial cells [109], suppressing PI3K/Akt and MAPK signaling, and enhancing cAMP/VASP pathways, highlighting the dual epigenetic and cardiovascular benefits of nut‐ and legume‐derived polyphenols [110].

**TABLE 8:** Effects of bioactive compounds in nuts and legumes on DNA methylation and platelet aggregation.

Bioactive compounds	Source	DNA methylation		Platelet aggregation/CVDs
		Study type and design	Suggested mechanism	
Catechins	Peanuts pistachios soybeans Green tea	In vitro study using prokaryotic SssI DNMT and human DNMT1	‐ Catechin, epicatechin, and EGCG → Modulate DNMT‐mediated methylation. ‐ EGCG is reported as a strong DNMT inhibitor (↑by Mg^2^⁺) [94].	Green tea catechins → ↓ PA (via Ca^2^⁺/IP_3_ pathway); antithrombotic potential [96].
		EGCG (5–50 µM): gene reactivation in cancer cells (PCR/ RT‐PCR/ Western blot)	‐ EGCG → Inhibits DNMT (*K_i_* = 6.89 µM), binds DNMT, demethylates CpG islands, reactivates silenced genes (e.g., p16, RARβ, MGMT) [95].	
Quercetin	Almonds Pistachios Walnuts	‐ In vitro (HL60 and U937 leukemia cells) ‐ In vivo (human tumor xenograft in mice)	‐ Quercetin downregulates DNMTs and STAT3. ‐ Leads to demethylation of BCL2L11 and DAPK1. ‐ Increases histone acetylation and reduces HDACs. ‐ Activates apoptosis‐related genes → enhances cell death [97].	‐ Quercetin inhibits platelet activation by binding GPIIb/IIIa, ‐ Reduces PDMPs. ‐ 3‐OH group enhances binding, helping preserve platelet integrity [99].
		In vivo study on male ICR mice ‐ Exposure: nickel sulfate ‐ Intervention: with or without quercetin co‐administration for 20 days.	Quercetin protected against nickel‐induced liver injury by: ‐ Lowering DNMT activity ‐ Demethylating Nrf2 ‐ Activating Nrf2/HO‐1 ‐ Suppressing inflammation and oxidative stress [98].	
Caffeic acid	Almonds Walnuts Cashews	‐ In vitro enzymatic assay ‐ Cell culture with HT29 cells ‐ Molecular docking analysis ‐ Animal study using Avy/a mice	‐ Caffeic acid phenethyl ester (CAPE) inhibits DNMT1 by competing with DNA ‐ Reduces DNA methylation ‐ Alters gene expression (e.g., agouti gene) [100].	Caffeic acid inhibits thrombin‐induced platelet activation (PA) without altering normal blood clotting by: ‐ Raising intracellular cAMP ‐ Activating VASP/IP3R pathway ‐ Suppressing ERK and Akt signaling ‐ Reducing intracellular calcium signals [102].
		‐ In vitro enzymatic assay ‐ Cell culture with breast cancer cells (MCF‐7, MDA‐MB‐231)	‐ Caffeic and chlorogenic acids inhibit DNMT via COMT‐mediated SAH formation [101].	
Gallic acid	Walnuts Hazelnuts Pecans	‐ In vitro studies on lung and oral cancer cells ‐ Compared anticancer effects of EGCG and gallic acid (GA) ‐ Analyzed gene expression profiles ‐ Tested PFOTE (polyphenol fraction of oolong tea extract) fermented with *Aspergillus sojae* for anticancer activity	‐ Gallic acid lowers DNMT1, DNMT3A, and DNMT3B ‐ Induces DNA demethylation ‐ Reactivates GADD45 expression ‐ Enhances cisplatin sensitivity, especially when combined with PFOTE treatment [103].	‐ Gallic acid directly inhibits thrombin by binding key residues (Ala230, Glu232, Ser235, Gly258, Gly260) ‐ Interaction via van der Waals forces stabilizes the thrombin–GA complex ‐ Reduces platelet activation (PA) [104].
		‐ In vitro study on human endothelial cells (EAhy926 and HBEC‐5i) ‐ Cells exposed to a combination of homocysteine, adenosine, and TNF.	‐ Gallic acid protects endothelial cells by restoring DNMT1 levels and inhibiting proteasome activity. ‐ Leads to cytoprotection and anti‐apoptotic effects [111].	
Genistein and Daidzein	Soybean Chickpea Pea Peanut Green soybean	‐ In vitro study on MCF‐7 and MDA‐MB‐231 cells ‐ Methods: Molecular modeling, methylation assays, qPCR ‐ Outcomes: Genistein effects	‐ Genistein reduces global DNA methylation ‐ Inhibits DNMT1 activity and blocks cytosine entry ‐ Reactivates tumor suppressor genes via promoter demethylation [105].	Genistein blocks PA via tyrosine kinase inhibition, similar to aspirin [112].
		Randomized, placebo‐controlled, double‐blind clinical trial on prostate cancer patients	‐ Genistein reduces DNA methylation and modulates gene expression ‐ Inhibits myelocytomatosis viral oncogene homolog (MYC) activity ‐ Promotes phosphatase and tensin homolog (PTEN) activity ‐ Influences epithelial‐to‐mesenchymal transition (EMT) and angiogenesis [106].	Genistein protects mice from doxorubicin‐induced cardiotoxicity by: ‐ Activating Nrf2/HO‐1 pathway ‐ Reducing oxidative stress, inflammation, and apoptosis [108].
		‐ In vitro study on prostate cancer cells: PC‐3, DU‐145, LNCaP ‐ Treatments: genistein, daidzein, E2, 5‐azacytidine, budesonide ‐ DNA methylation assessed by methyl‐profiler and qPCR	‐ Genistein, daidzein, and E2 induce DNA demethylation via ERβ binding, similar to 5‐azacytidine ‐ Budesonide increases DNA methylation, likely through modulation of DNMT and HDAC activities [113].	‐ Genistein and daidzein inhibit platelet aggregation ‐ Reduce NO and TNF‐α production in macrophages ‐ Lower MCP‐1 levels in endothelial cells ‐ Genistein exhibits stronger effects, indicating potential cardiovascular protection [109].
		‐ A case‐control study in 20 women ‐ Intervention group: 10 women consumed soymilk for 5 days ‐ DNA methylation analyzed by MethylCap‐seq and LC‐MS	Soy isoflavones may inhibit DNMTs and HDACs, but short‐term intake showed no significant methylation changes [107].	Daidzein inhibits collagen‐induced platelet activation by: ‐ Reducing TXA2 and granule release ‐ Suppressing PI3K/Akt and MAPK pathways ‐ Increasing cAMP levels and VASP phosphorylation [110].

Overall, the results from in vitro and animal studies provide important mechanism support for the role of bioactive compounds in nuts and legumes in modulating diverse signaling cascades, including Ca^2^⁺/IP_3_, PI3K/Akt, MAPK, cAMP/VASP, and Nrf2/HO‐1 pathway. However, the translational applicability of these findings to humans remains uncertain, as clinical data, particularly from short‐term interventions, have yielded inconsistent results. This emphasizes the necessity for precisely designed, long‐term RCTs to validate their epigenetic and cardiovascular effects in human populations. Moreover, interindividual variability in the specific genes related to demethylation, transcriptional reprogramming, and gene expression, associated with factors such as bioavailability, metabolic transformation, and matrix interactions of nuts and their bioactive compounds, may critically influence the observed outcomes. While current evidence relates several bioactive compounds abundant in nuts and legumes to DNA methylation and favorable cardiovascular outcomes, existing limitations preclude definitive conclusions regarding causality.

**FIGURE 4:**
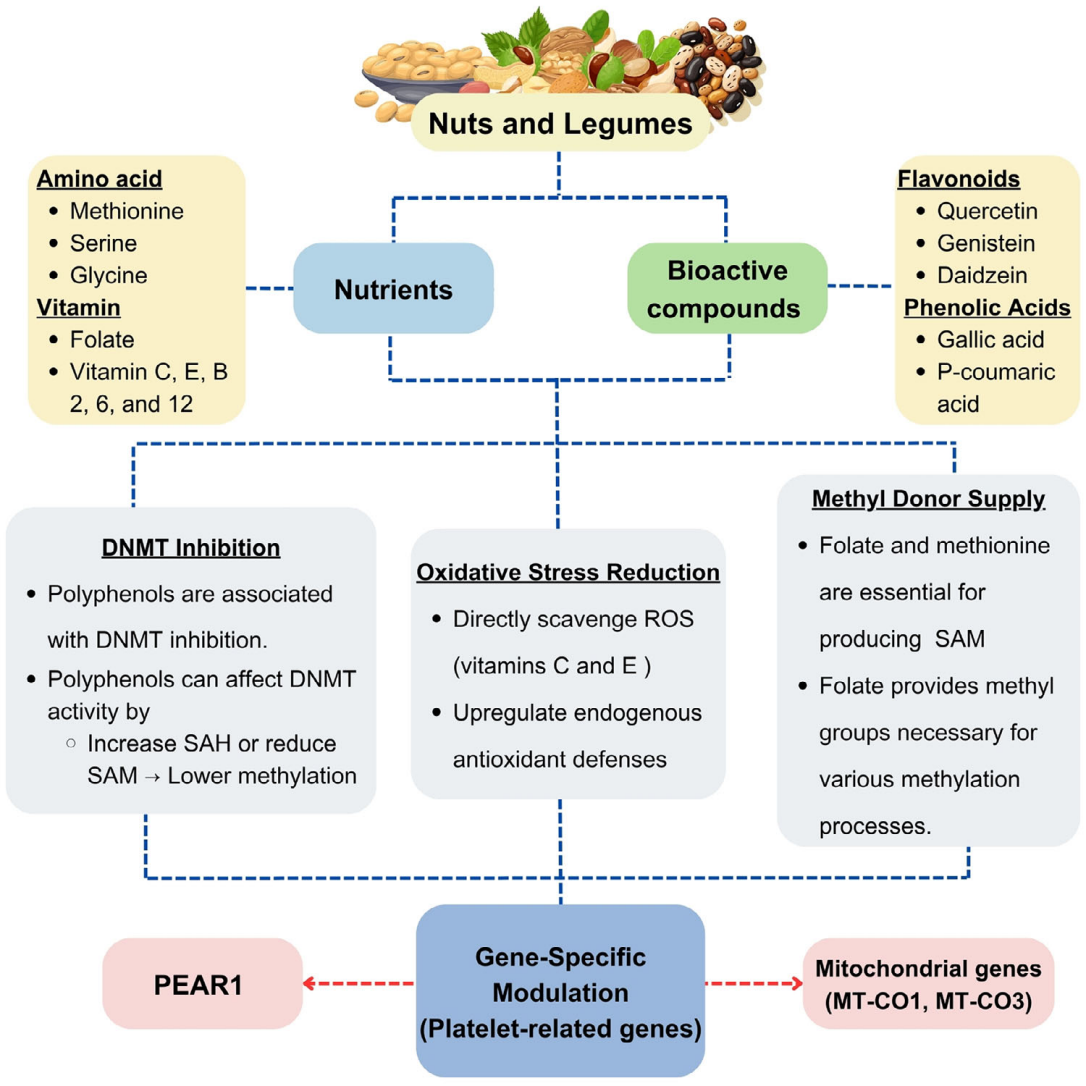
Nutrients and bioactive compounds in nuts and legumes function through multiple mechanisms to influence platelet function via epigenetic pathways (created with CAVA.com, by Siwaphorn Chaimati).

Polyphenols are related to modulating metabolism, leading to increased levels of S‐adenosylhomocysteine, which is known to inhibit DNMTs [114]. Furthermore, polyphenols have been shown to influence histone modifications related to DNA methylation in the regulation of gene expression [115]. Dietary antioxidants are crucial in mitigating oxidative and DNA damage, supporting normal methylation and histone modifications [116]. Research suggested that oxidative stress affects chromatin structure and DNA methylation, emphasizing the importance of antioxidants in maintaining epigenetic stability. These antioxidants work through several mechanisms: they directly scavenge ROS, with antioxidants like vitamins C and E neutralizing free radicals to prevent DNA damage [117]; polyphenols related to upregulate endogenous antioxidant defenses [118], with specific dietary components enhancing the body's antioxidant systems, thereby limiting cellular damage and promoting integrity [119]; and some antioxidants modulate DNA repair mechanisms, such as vitamin C, which has been shown to help in repairing oxidative DNA damage in vivo [120]. Furthermore, folate and methionine are vital for producing SAM, a key methyl donor in DNA methylation, with folate specifically providing methyl groups necessary for various methylation processes [121]. Disruptions in this pathway, such as folate deficiency, can result in decreased SAM levels and aberrant DNA methylation patterns, potentially resulting in altered gene expression and increased disease risk [122]. These epigenetic changes extend to genes such as *PEAR1* and mitochondrial genes (*MT‐CO1*, *MT‐CO3*), which influence platelet function and thrombosis [123]. So, nutrients and bioactive compounds found in nuts and legumes utilize various mechanisms to regulate platelet function through epigenetic pathways shown in Figure 4.

In previous studies, nuts such as walnuts, almonds, and pistachios are rich in polyphenols, which have been associated with anti‐PA and epigenetic effects. Almonds contain flavonoids, such as quercetin and isorhamnetin, which regulate eNOS, enhance NO production, and stabilize the methylation of platelet‐related genes [44, 124]. By improving NO production, these flavonoids reduce oxidative stress, a key disruptor of DNA methylation, and enhance the methylation stability of platelet‐related genes. Pistachios, abundant in catechins and proanthocyanidins [46], are associated with downregulating inflammatory pathways, inhibiting DNMT activity, and modulating histone acetylation, thereby reducing PA [125]. Legumes, such as soybeans, lentils, chickpeas, and black beans, are rich in nutrients and phytochemicals that regulate DNA methylation. Isoflavones such as genistein and daidzein in soybeans are related to inhibiting DNMT activity, potentially leading to the hypomethylation of genes linked to PA and inflammation [105, 126, 127]. Folate, abundant in lentils and chickpeas, supports one‐carbon metabolism and SAM synthesis, helping maintain normal methylation patterns and preventing platelet hyperactivation [128]. In addition, phenolic acids such as gallic acid in chickpeas suppress thromboxane A2 synthesis, improve platelet function, and reduce CVD risk [129].

In addition to their potential epigenetic effects, particular polyphenolic compounds have been investigated for their roles in modulating platelet activation pathways. For example, in experimental models, quercetin has been reported to interact with GPIIb/IIIa receptors and reduce the release of platelet‐derived microparticles, which may contribute to platelet membrane integrity [99]. Similarly, catechins such as EGCG have been observed to reduce PA, possibly related to modulation of intracellular calcium signaling and inositol triphosphate pathways involved in fibrinogen binding and thrombus formation [96]. However, these findings are primarily based on in vitro or animal studies, and further research is needed to clarify their relevance to human health.

Collectively, current evidence suggests that bioactive compounds present in nuts and legumes may influence PA and thrombosis through both direct biochemical mechanisms and potential epigenetic modulation of gene expression involved in oxidative stress responses, endothelial function, and platelet signaling pathways [96, 99, 103, 105, 123]. Although these dual mechanisms have been suggested to contribute to the cardioprotective effects of plant‐based diets, further research, especially in human populations, is needed to confirm these associations.

## 5 Conclusion and Future Research Suggestions

Nuts and legumes have many reports on promoting cardiovascular health by reducing PA related to epigenetic mechanisms, particularly DNA methylation. Their rich composition of bioactive compounds, like polyphenols, flavonoids, phenolic acids, and nutrients, supports favorable epigenetic modifications, particularly in genes, such as *PEAR1*, which regulate platelet function. Regularly consuming these foods is associated with improved lipid profiles, reduced oxidative stress, and lowered inflammation, highlighting their potential to mitigate CVD risk. While recent evidence suggests beneficial associations between nuts and legumes intake, altered DNA methylation patterns, and improved cardiovascular health markers, such as PA, most findings are based on in vitro, in vivo, observational, or short‐term interventions and thus cannot confirm a causal relationship.

However, several important aspects require further study. Future research should investigate varieties of nuts and legumes, such as macadamia nuts, mung beans, and pigeon peas, which may contain unique bioactive profiles but remain inadequately characterized, as reported in the current study. Additionally, the long‐term effects of these foods on epigenetic changes and platelet function across diverse populations differing in genetic backgrounds, metabolic phenotypes, and lifestyle factors should be systematically considered. Moreover, it should also assess the potential synergistic effects of nuts and legumes consumption when combined into more general dietary patterns like Mediterranean, DASH diets, or other diets/nutrients defined as antiplatelet agents. Furthermore, RCTs are necessary to define optimal and develop the recommended intake levels, assess compound bioavailability, and provide relations between specific dietary components and epigenetic markers associated with platelet activity changes.

Appearing omics technologies, such as genomics, epigenomics, metabolomics, transcriptomics, and nutrigenomics, effectively identify biomarkers and clarify the molecular mechanisms by which dietary compounds affect health outcomes. These approaches should support our understanding of DNA methylation and other epigenetic mechanisms, including histone modifications and noncoding RNAs, which may also be modulated by nutrients and phytonutrients of nuts and legumes. Moreover, promoting the development of functional foods enriched with these compounds may provide innovative, targeted approaches to reduce CVD risk. Personalized nutrition based on individual epigenetic profiles should also improve prevention. Combining various nuts and legumes into the daily diet represents an advantageous, natural, and sustainable approach to reducing the risk of CVD and encouraging long‐term health.

## Funding

The authors have nothing to report.

## Conflicts of Interest

The authors declare no conflicts of interest.

## Data Availability

Data sharing not applicable to this article as no datasets were generated or analyzed during the current study.
